# Development of Porous Polyvinyl Acetate/Polypyrrole/Gallic Acid Scaffolds Using Supercritical CO_2_ as Tissue Regenerative Agents

**DOI:** 10.3390/polym14040672

**Published:** 2022-02-10

**Authors:** Diego Valor, Antonio Montes, Antonio Cózar, Clara Pereyra, Enrique Martínez de la Ossa

**Affiliations:** Department of Chemical Engineering and Food Technology, Faculty of Sciences, University of Cadiz, International Excellence Agrifood Campus (CeiA3), Campus Universitario Río San Pedro, Puerto Real, 11510 Cadiz, Spain; antonio.montes@uca.es (A.M.); antonio.cozaralmagro@alum.uca.es (A.C.); clara.pereyra@uca.es (C.P.); enrique.martinezdelaossa@uca.es (E.M.d.l.O.)

**Keywords:** polypyrrole, polyvinyl acetate, scaffolds, supercritical CO_2_ foaming, drug delivery

## Abstract

Scaffolds are advanced devices employed in tissue engineering, as they are intended to mimic the characteristics of extracellular matrices. In this respect, conjugated materials are gaining relevance in the manufacturing of the foams used for therapeutic scaffolds, since they can provide certain properties that are missing in the other polymers used to form the scaffolds. This work has, therefore, focused on the development of functional scaffolds formed by conjugated-non-conjugated polymers such as polyvinyl acetate and polypyrrole, impregnated with gallic acid as the model drug and produced by means of a supercritical CO_2_ foaming/impregnation process. The effects from a series of parameters such as pressure, temperature, depressurization rate, and contact time of the scaffold production process have been determined. The impregnated foams have been characterized according to their morphology, including their porosity and expansion factor, their drug loading and delivering capabilities, and their mechanical and electrical properties. The characterization of the experiments was carried out using scanning electron microscopy, liquid displacement, in vitro release, electrochemical impedance spectroscopy, and compression techniques. The results from our tests have revealed a considerable influence of all the input variables studied, as well as relevant interactions between them. Values close to 35% porosity were obtained, with a drug release of up to 10 h with a fast initial release. The best operating conditions were 353 K, 30 MPa, 0.5 MPa/min depressurization rate, and 1 h contact time. By means of the supercritical foaming/impregnation technique, scaffolds with potential in tissue engineering due to their studied properties were obtained.

## 1. Introduction

Tissue engineering is a currently emerging scientific discipline. It focuses on the regeneration of damaged or destroyed tissues/organs in living organisms, and substantial improvements have been achieved over the last decade [1,2]. Given this state of things, the biomaterials that are being used for wound healing and tissue regeneration have attracted the interest of the scientific community. Although human skin can self-repair and recuperate its structural and functional integrity, wound care is still quite necessary to prevent certain problems such as infections, pain, exposure of an open area, or scar formation, among others [3,4]. One of the key properties of human tissues is its conductivity, since human cells produce an electric field as part of the self-healing process [5]. It is, therefore, essential to use conductive biomaterials that support the activity of the electrically responsive cells so that the healing process can be accelerated [6].

Conjugated conductive polymers are presented as an effective novel technology that can meet and provide the necessary conductivity characteristics that allow to transfer the electrical signals required by the healing cells [7,8]. Because of their biocompatibility, polypyrrole (PPy), polyaniline (PANI), and poly(3,4-ethylenedioxythiophene) (PEDOT) are some of the polymers that are most commonly used for these purposes [9]. PPy, particularly, is a well-known polymer among the many conductive ones, since it is easy to synthesize, exhibits excellent conductive properties, and has favorable redox characteristics [10,11,12]. PPy has been often used for medical purposes as a conductive element in films [13,14,15], aerogels [8], membrane dressing [12,16], or nanofibers [17] among other applications.

Unfortunately, conductive polymers do not hold a number of characteristics that are quite relevant when intended for tissue regeneration purposes, such as durability, strength, or solubility. In other cases, their shortcomings are associated to their complex handling and processing [18]. Therefore, and in order to overcome these inconveniences, conductive polymers are frequently combined with other non-conductive polymers, whether natural, such as alginate or chitosan, or synthetic polymers, such as polyvinyl alcohol (PVA), poly (lactic-co-glycolic acid) (PLGA), polylactide (PLA), polyvinylpyrrolidone (PVP), polycaprolactone (PCL), and/or polyvinyl acetate (PVAc), among others [19,20].

Vinyl polymers such as PVAc, PVA, or PVP are widely used in biomedical applications, as suitable scaffolds for tissue regeneration and/or to carry and deliver drugs thanks to their biocompatibility. PVAc, in particular, is an inert polymer that presents the advantage of not inducing any deleterious reactions in living tissue, which makes it suitable for the synthesizing of vascular grafts [21].

The properties exhibited by polymer composites intended to be used for tissue engineering such as porosity, fibrosity, permeability, and mechanical stability must allow their successfully mimicking extracellular matrices (ECM) [22]. In this sense, the scaffolds that are made of polymeric biomaterials usually provide the structural support required for cell adhesion and for the subsequent tissue regeneration [23]. Besides their mechanical function, they can also be used as devices that gradually delivery the desired drugs into the area surrounding the target tissues and for a certain period of time [24,25].

With regard to scaffold manufacturing, numerous techniques have been developed over the years for the processing of biodegradable polymers to produce different types of scaffolds. Solvent casting, particle leaching, fusion molding, extrusion, or freeze drying are some of the conventional techniques that have been used until present. Other advanced techniques are electrospinning, fused deposition modeling, 3D printing, selective laser sintering, and bioprinting [26]. However, many of these processes cannot produce a polymeric matrix with the morphology, pore size, and high interconnectivity that would be desirable [27]. Moreover, the high temperatures required by many of these production techniques and the use of organic solvents cannot only result in accelerated polymer degradation, but the bioactive compounds that should be ideally incorporated to the scaffolds’ pores and surface may also become deteriorated [28].

Supercritical carbon dioxide (scCO_2_) foaming presents certain specific benefits as an environmentally friendly method for the production of porous scaffolds. Moreover, this process does not require the use of organic solvents and can be operated at a relatively low temperature [28]. Due to the low viscosity and high diffusivity of scCO_2_, it can easily penetrate the polymeric matrix, hence favoring the plasticization process. The system remains saturated over the contact time, and the depressurization stage, the carbon dioxide supersaturation in the polymer matrix causes the nucleation and growth of the porous cells within the polymeric matrix [24]. Numerous studies on the formation of polymeric scaffolds by supercritical foaming processes have been carried out. Porous scaffolds, for example, have been generated from biodegradable synthetic polymers (PLA and PLGA) in combination with a bioactive substance, creating controlled drug-releasing systems based on the foaming/impregnation process that takes place when using scCO_2_ [29].

The aim of this work is to develop tissue repair and drug delivery systems by combining conductive and non-conductive polymers. Gallic acid (GA) has been used as the model drug for its anti-inflammatory, antioxidant, and cardiovascular protective properties [30,31]. The authors have evaluated the influence of the main process variables, such as pressure, temperature, depressurization rate, and impregnation/foaming time on the polymer expansion factor, electrical conductivity, mechanical resistance, and textural properties, as well as the gallic acid release profile of the resulting scaffolds.

## 2. Materials and Methods

### 2.1. Materials

Polyvinyl acetate (PVAc) and polypyrrole (PPy) were provided by Sigma–Aldrich (Madrid, Spain). Gallic acid (C_6_H_2_(OH)_3_CO_2_H) (GA) was purchased from Sigma–Aldrich (Madrid, Spain). Potassium chloride (KCl), disodium-hydrogen phosphate (Na_2_HPO_4_), potassium dihydrogen phosphate (KH_2_PO_4_), and sodium chloride (NaCl) were purchased from Panreac Applychem (Barcelona, Spain). CO_2_ with a minimum purity of 99.8% was supplied by Linde (Barcelona, Spain).

### 2.2. Foaming and Impregnation Process with scCO_2_

**Figure 1:**
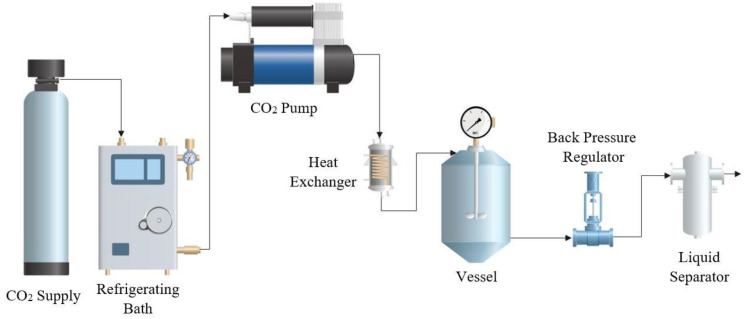
Diagram of the SSI pilot plant used for the experiments.

The polymer foaming and impregnation experiments were carried out in a pilot plant (Iberfluid, Barcelona, Spain) as schematically shown in Figure 1. The apparatus is equipped with a thermal bath and a high-pressure pump that allows the CO_2_ to be injected in liquid state. The temperature is controlled by an electric heat exchanger, located just before the CO_2_ inlet to the 500 mL foaming vessel. The required pressure of the entire system is adjusted by means of an automatic backpressure regulator valve. Finally, the system is equipped with a micrometric valve that allows to adjust the vessel depressurization rate and vents the CO_2_ through a cyclonic separator.

Firstly, each sample containing 200 mg of PVAc, 60 mg of PPy, and 30 mg of gallic acid was manually mixed in an aluminium foil holder, which was then placed in the foaming vessel. Then, the scCO_2_ was pumped into the vessel until the desired conditions for the foaming/impregnation process were reached. The polymer mixture was kept in contact with the CO_2_ for the set time to allow it to penetrate the polymer and cause the plasticization effect. Then, the system was depressurized at the desired controlled rate by means of the automatic back-pressure regulator valve, which resulted in the foaming and impregnation of the final polymeric structure.

A total of 12 experiments were carried out in order to study the effect of parameters such as pressure, temperature, depressurization rate, and contact time on the formation process of porous foams. This study was carried out in the range of 10–30 MPa of pressure (P), temperatures (T) of 313–353 K, and a foaming/impregnation time (t) of 1–4 h. In the case of temperatures, the values selected for the tests were chosen according to the melting point of the PVAc (333 K), working below and above this point. Once the process time had finished, the output valve was opened to vent the CO_2_ in a depressurization rate (Dr) ratio of 0.5–2 MPa/min. Run 12 was carried out in order to observe the repeatability of the process by reproducing the same conditions as Run 6 in terms of porosity, expansion factor or mechanical properties. All the performed experiments can be seen in detail in Table 1.

### 2.3. Scanning Electron Microscopy (SEM)

The morphology of the impregnated scaffolds was examined by scanning electron microscope (SEM). A Nova NanoSEM 450^TM^ (Elecmi, Zaragoza, Spain) with an accelerating voltage of 30 kV was used. In order to obtain a correct viewing of the samples, a cross-section of each polymer coated with a 10 nm film of gold prior to analysis was selected.

### 2.4. Volumetric Expansion of Samples

The volume increase of the resulting scaffolds was evaluated by calculating their expansion factor [32]. The expansion factor was determined according to the following expression (Equation (1)):

(1) $$\mathrm{Expansion}\;\mathrm{Factor}=\frac{\mathrm{Final}\;\mathrm{volume}}{\mathrm{Initial}\;\mathrm{volume}}$$

### 2.5. Porosity Estimation

The porosity of the samples was determined by calculating the ratio between the holes in the produced samples by pores and the total volume of the polymer. The measurement of this parameter is given by the movement of a fluid inside the polymer [32,33]. The selected liquid for the porosity analysis was ultrapure water, because it does not damage the structure of the polymeric scaffold and is able to penetrate correctly between the pores of the used material.

For the calculation, a portion of the samples was immersed in a given volume (V1) until it was completely covered; subsequent to this, the resulting volume was then measured (V2). Finally, the scaffold was removed from the liquid, and the residual remaining volume of water was measured (V3). Thus, an estimation of porosity was achieved according to Equation (2):

(2) $$\mathrm{Porosity}\;\left(\%\right)=\frac{\mathrm{volume}\;\mathrm{of}\;\mathrm{holes}}{\mathrm{total}\;\mathrm{volume}\;\mathrm{of}\;\mathrm{sample}}=\frac{V1-V3}{V2-V3}\cdot 100$$

### 2.6. Impregnation Percentage and Release of Gallic Acid

In order to determine the gallic acid’s release profiles, GA-impregnated PVAc/PPy foam samples were weighted (40 mg) and suspended in 25 mL of 0.05 M phosphate buffer solution (PBS) stirred at 200 rpm at constant 37 °C. The PBS (1 L) was prepared mixing 18.4 mL of monobasic potassium phosphate with 31.6 mL of dibasic potassium phosphate in distilled water at pH 7.4. Based on the calculations reported by Zhu et al. [34], the release of gallic acid into the solution was measured after 5, 15, 30, and 60 min, and then every hour by determining its concentration based on an aliquot (3 mL) of the release solution measured by means of a UV-VIS spectrophotometer at λ = 270 nm. After the total determination of the impregnated GA, the percentage of PPy present in the sample was calculated by weighting difference according to the initial used weight.

### 2.7. Electric Properties

Conductivity analysis was performed by calculating resistivity, as impedance, that is defined as the inverse of conductivity. Thus, the lowest measured impedance value the highest level of conductivity of the scaffolds. Electrochemical impedance spectroscopy presents the signal as a function of frequency at a constant potential, generating analyzable impedance measurements at each frequency. The generated scaffolds were analyzed using a Solartron 1260 impedance spectroscope (AMETEK Scientific Instruments, Oak Ridge, TN, USA). The samples were cut into 15 mm^3^ portions and were subjected at room temperature to 1 V alternating current at frequencies ranging from 1.00 Hz to 1.00 kHz.

### 2.8. Mechanical Resistance

A number of compression tests were carried out in order to determine the mechanical properties of the final scaffolds. The deformation of 15 mm^3^ cube samples subjected to stress, was measured by means of an Criterion C45 tester (MTS, Eden Prairie, MN, USA). The mechanical resistance of the scaffolds was quantified by Young modulus (E), calculated as the slope of the elastic region of the stress–strain curve obtained from the compression tests [35]. The maximum force applied to the scaffolds was 10 kN, where the total stress was 60% at a compression speed of 0.01 mm/s.

## 3. Results and Discussion

### Foaming and Impregnation Experiments

**Figure 2:**
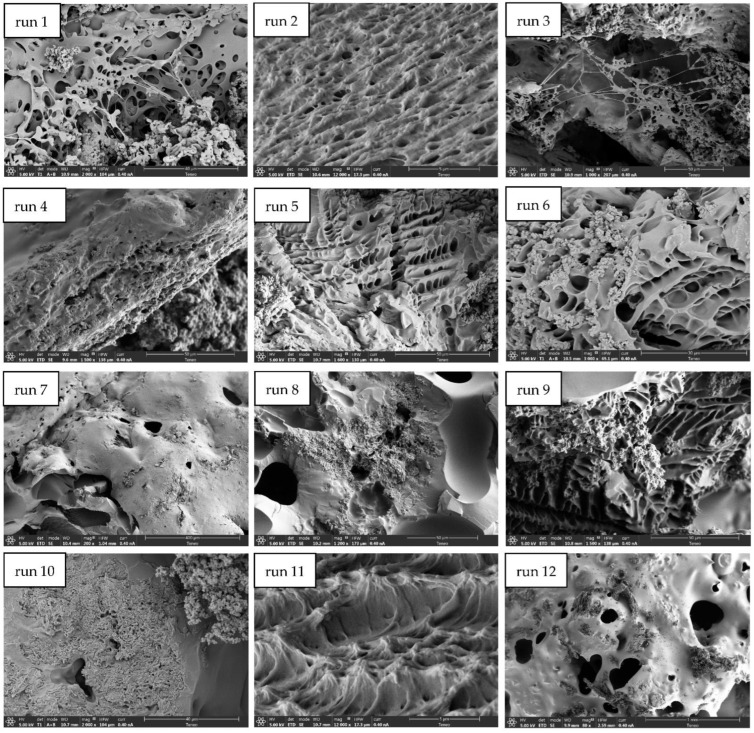
SEM images of PVAc/PPy produced scaffolds.

All tests exhibited visually an inhomogeneous impregnation along the structure of the PVAc. Some representative SEM images of the produced PVAc/PPy composites are shown in Figure 2.

As can be observed from the SEM images, many of the structures obtained exhibited irregular porosity. It can be clearly observed that part of the PPy and the GA were impregnated both inside and outside the porous nuclei of the scaffolds produced by runs 1, 3, 8, and 12. The accumulation of impregnated compounds on some of the samples’ surface does not allow the clear observation of the arrangement achieved inside the pores of the scaffold. Nevertheless, evident differences could be observed with regard to foaming and impregnation results when comparing the results obtained from the different tested conditions.

**Figure 3:**
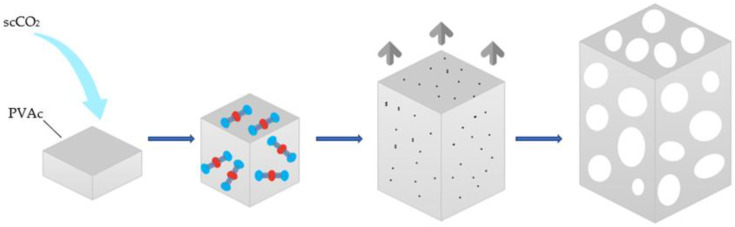
Schematic diagram of the formation of the porous structures by supercritical CO_2_.

Porosity is one of the most important characteristics in polymeric-foam production. When intended to be used for tissue engineering, porous structures should facilitate cell seeding and cell penetration and allow the distribution of other compounds inside the scaffolds [36]. Porous foams should also provide an empty space that allows the vascularization and regeneration of the living tissue. Throughout the scCO_2_ foaming process, CO_2_ diffuses and dissolves into the polymer matrix and plasticizes it, resulting in a significant Tg (glass transition temperature) drop and thus causing the polymer to melt. Bubble nucleation would occur and subsequently pores would grow. At the same time, as the polymer is drained of CO_2_, the Tg increases inversely and the plasticization effect weakens, leading to vitrification of the matrix, which in turn inhibits pore formation. When the vitrified polymer becomes too rigid to expand, foaming is complete, and the voids occupied by the gas bubble are rigidly preserved, thus creating an expanded porous scaffold [37]. A schematic diagram of the process is shown in Figure 3.

**Figure 4:**
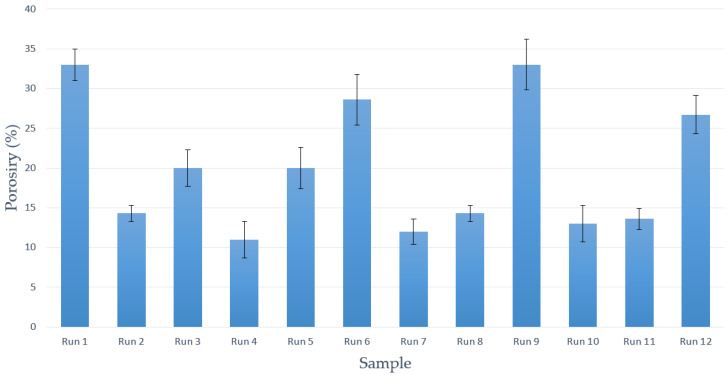
Porosity percentages of the final scaffolds.

The porosity percentages corresponding to the foams from each experimental run are shown in Figure 4. The experiments that achieved the highest porosity percentages, over 25%, were Runs 1, 6, and 9. The main conclusion reached is that shorter contact times with CO_2_ led to a higher porosity percentage of the foams under all the studied conditions. Previous studies had already reported that by varying the soaking time the properties of the resulting polymeric foams would largely be affected [38]. Gualandi et al. [39] concluded that, under certain conditions, by increasing the soaking time of the polymer in CO_2_, a decrease in the average porosity and pore size would take place. Higher concentrations of CO_2_ dispersed into the polymeric matrix may also lead to a higher nucleation density that would result in structures with smaller pores, which represents a disadvantage when trying to impregnate compounds such as PPy or GA. In general, higher porosity values were obtained when 313 K was used. This temperature seems to be enough to melt the PVAc/Ppy mixture and to promote the dissolution of the CO_2_ with the subsequent generation of bubbles and pores inside the foams [33]. On the other hand, neither pressure nor depressurization rate values seemed to affect the porous formation process, and relatively high porosity percentages were achieved under all the tested conditions.

**Figure 5:**
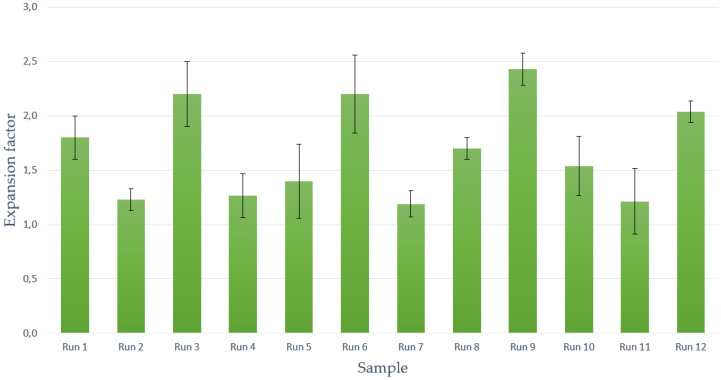
Expansion factor of obtained scaffolds.

Another parameter that has been studied is the volumetric expansion factor reached after the foaming/impregnation process, which is closely related to the porosity of the final composites. Polymeric scaffolds, which are intended for use in tissue engineering, must provide structural support for exogenously applied cells to grow, attach, or migrate. To achieve this goal, cell attachment sites are needed, a porous structure with interconnectivity for the diffusion of possible nutrients or cell migration. These requirements are closely related to the expansion of the polymer, which provides the required porosity. On the other hand, they must have appropriate mechanical properties to fill the empty space in the defect where it is supposed to simulate native tissue. One of the important parameters when filling these voids is the expansion factor [23]. The expansion factor corresponding to the scaffolds obtained from each run are shown in Figure 5. As expected, the scaffolds with higher porosity percentages (Runs 1, 6, and 9) are among the ones exhibiting higher expansion factors (over 1.5). Regarding the PVAc volume growth when exposed to supercritical conditions, it can be observed that the use of lower depressurization rates favors such increase (Runs 3, 8, 9, and 10). Previous studies had already pointed out that by decreasing the depressurization rate, the samples’ volume growth would be increased due to a lower CO_2_ concentration, which results in larger cells’ diameter and a greater overall expansion [40]. In contrast, other variables, such as pressure, temperature, or contact time had no apparent effect on the volumetric growth of the foams under the conditions tested in this work. Comparing the best results in terms of porosity, which are around 30% and 35% with the literature, there are studies with PCL scaffolds applied to chondrogenesis in which the average porosity used is 30% [41] or applications in osteogenesis with porosities close to 35% [42]. Thus, the authors consider that the porosity data obtained may be acceptable for some of the applications in tissue engineering.

Additionally, the impregnation quantity of PPy and GA was studied. Both compounds are not subjected to the foaming process by supercritical CO_2_, where PVAc was foamed. To achieve this objective, a total release of GA in a PBS solution was performed to determine, by UV spectroscopy, the total released amount and therefore impregnated into the scaffolds. Subsequently, the quantity of impregnated PPy was calculated by weighting the difference between the collected sample, the initial total quantity that was deposited, and the impregnated GA. The corresponding data are shown in Table 2.

**Figure 6:**
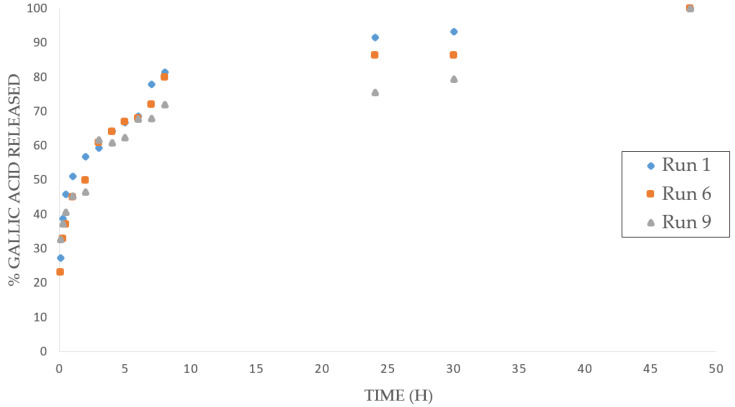
Release profile of gallic acid in Runs 1, 6, and 9.

In general, the samples with a larger porosity ratio were the only ones that presented a large number of compounds impregnated, both GA and PPy. Figure 6 shows the GA release profiles into the PBS solution of the samples with the highest impregnation percentages (Experiments 1, 6, and 9), at over 30%. It can be observed that the three samples exhibit a very similar release profile, with a rapid burst release of the drug at the beginning of the test, that slows down after 5 h and with the longest releasing period stretching up to 2 days. A gradual and relatively uniform release was therefore achieved, in terms of similar release being obtained in samples with higher porosity and impregnation, which is a crucial factor for certain biomedical applications such as implants for tissue regeneration.

On the other hand, the foaming/impregnation process was used to introduce the PPy into the scaffold structure and surface to give the desired electrical properties for their use in tissue engineering. It should be noted that, in experiments that did not show a large expansion factor or high porosity, relatively high percentages of PPy impregnation were achieved. In that case, probably part of PPy will be located on surface of polymer due to the difficulty to penetrate into a polymer. This could indicate a reduced distribution in the structure and, hence, in the electrical conductivity.

Regarding the mechanical and electrical properties of the final scaffolds, their impedance and mechanical strength data are presented in Table 3.

The conductivity of the scaffold was indirectly measured by impedance spectroscopy to validate that the impregnated PPy provided electrical properties to the foams, since impedance is defined as the effective resistance of an electrical circuit or component to alternating current. A lower impedance to current flow was determined in the Runs 2, 3, 4, 5, and 9; thus, superior electrical conductive properties were reached in these runs. In this case, the obtained values are highly related to the percentage of impregnated PPy in the scaffolds, with the samples with more impregnated conductive polymer showing better conductive conditions. Most of these runs were conducted at low temperatures, high pressures, and low CO_2_ depressurization rates. Among them, Run 2, which was carried out at low pressure (100 bar) and a high depressurization rate (2 MPa/min), showed the lowest conductivity, thus demonstrating the influence of the studied parameters on the electrical behavior of the composites. The differences in impedance values between polymers with similar impregnation percentage may also be due to the heterogeneous impregnation of PPy in the foam structure previously discussed.

Other requirements for scaffolds to be used in tissue engineering are supplying the desired form and mechanical support to the tissue defect and giving the rigidity and stiffness to the engineered tissues [23]. The strength that the scaffold should provide varies according to the type of tissue to be treated and ranges from 2 and 6000 MPa depending if it is a fibrous or a mature bone tissue, respectively [43]. Table 3 shows the Young’s modulus and stress peak values for each of the performed tests. Young’s modulus is a parameter that reveals the behavior of an elastic material as a function of the applied force to it and the consequent increase or decrease in the length of that material, thus meaning a higher elasticity with the increase of the module. The porosity and the elastic behavior of the materials obtained are highly related, in an inverse proportion, as demonstrated by the highest values of Young’s modulus in the tests that showed the lowest porosity (Runs 7, 10, and 11). In general, lower expansion factor and porosity appears to be related with higher mechanical resistance. However, in the case of Tests 6 and 9, where high porosity was obtained, a relatively high young modulus (in the order of 10 MPa) was obtained. The rest of the studied parameters do not have a clear tendency regarding the mechanical properties, obtaining disperse values in both levels.

Furthermore, the repeatability of the supercritical foaming process was verified by carrying out Run 12, which was performed under the same conditions as Run 6 in order to compare the results (353 K temperature, 10 MPa pressure, 2 MPa/min depressurization rate, and 1 h contact time). Differences below 10% were obtained for the studied response variables, i.e., porosity, expansion factor, conductivity, and Young’s modulus. According to the authors’ criteria, the repeatability of the process has been confirmed for the tested conditions.

## 4. Conclusions

Polyvinyl acetate/polypyrrole/gallic acid scaffolds have been successfully produced using a one-step supercritical CO_2_ foaming/impregnation process. Foam structures based on the non-conductive polymer (polyvinyl acetate) were produced while a conductive polymer (polypyrrole) was impregnated to provide the said structures with the required electrical conductive properties for medical usage. Moreover, gallic acid was also incorporated to the foam in order to obtain functional scaffolds. In general, the scaffolds that had been formed at low temperature and using a short contact time presented a greater expansion factor and porosity ratio, obtaining maximum porosity values of around 35% and expansion factors greater than 2. The largest PPy loads corresponded to the scaffolds that exhibited a higher porosity percentage and a greater expansion factor. It has been demonstrated that the amount of PPy inside the samples is an important factor regarding the electrical properties that PPy provides to the scaffolds. Thus, the samples where the PPy was mostly located on the foams’ surface failed to exhibit the required conductive capabilities. On the other hand, the drug-releasing capacity of the developed structures has been tested using gallic acid as the model substance, and up to 2 days releasing periods were registered in the best cases. The mechanical strength and elasticity of the composites were also determined and satisfactory results, in terms of their suitability for tissue-regenerating applications was confirmed, with higher strength corresponding to the less porous samples. It has been demonstrated that supercritical CO_2_ foaming has the capacity to produce foam scaffolds based on PVAc and polypyrrole that can potentially be used for medical purposes such as tissue regeneration and/or the delivery of therapeutical substances. Further research should be conducted to determine the optimal processing conditions for the production of scaffolds for each specific application.

## Figures and Tables

**Table 1:** Summary of the foaming experiments and values for the variables studied.

Runs	T (K)	P (MPa)	Dr (MPa/min)	t (h)
1	313	10	2	1
2	313	10	2	4
3	313	30	0.5	4
4	313	30	2	1
5	313	30	2	4
6	353	10	2	1
7	353	10	2	4
8	353	10	0.5	4
9	353	30	0.5	1
10	353	30	0.5	4
11	353	30	2	1
12 *	353	10	2	1

* reproducibility test.

**Table 2:** Summary of GA and PPy impregnated quantities in the developed scaffolds.

Run	Impregnated GA (mg)	Impregnated PPy (mg)	Total GA + Ppy Impregnated (%)
1	5.04	30.84	33.08
2	2.76	20.43	22.42
3	1.21	11.42	12.13
4	0.64	7.95	8.74
5	2.35	20.87	21.03
6	7.83	29.89	33.22
7	4.51	15.09	16.22
8	4.77	12.76	12.61
9	7.55	31.58	34.24
10	2.13	20.43	21.98
11	0.51	20.51	20.39

**Table 3:** Summary of impedance (Ω) and mechanical strength (Young’s modulus, MPa) results of scaffolds.

Runs	I ^1^ (Ω)	PS ^2^ (MPa)	E ^3^ (MPa)
1	4.17 · 10^8^	7.28	3.54
2	4. 09 · 10^8^	25.03	3.65
3	5.20 · 10^5^	8.82	0.80
4	5.50 · 10^5^	3.51	10.30
5	3.22 · 10^5^	8.09	12.55
6	4.52 · 10^8^	13.00	8.68
7	3.52 · 10^8^	32.12	29.94
8	3.39 · 10^8^	8.49	5.27
9	4.03 · 10^6^	18.64	10.45
10	4.03 · 10^8^	28.64	20.78
11	4.33 · 10^8^	3.79	15.67
12	3.68 · 10^8^	17.23	7.94

^1^ I = Impedance; ^2^ PS = Peak Stress; ^3^ E = Young Modulus.

## Data Availability

The data presented in this study are available on request from the corresponding author.
